# Antidepressant and Anxiolytic Effects of Medicinal Cannabis Use in an Observational Trial

**DOI:** 10.3389/fpsyt.2021.729800

**Published:** 2021-09-09

**Authors:** Erin L. Martin, Justin C. Strickland, Nicolas J. Schlienz, Joel Munson, Heather Jackson, Marcel O. Bonn-Miller, Ryan Vandrey

**Affiliations:** ^1^Department of Neuroscience, Medical University of South Carolina, Charleston, SC, United States; ^2^Behavioral Pharmacology Research Unit, Department of Psychiatry and Behavioral Sciences, Johns Hopkins University School of Medicine, Baltimore, MD, United States; ^3^Department of Psychology, University at Buffalo, Buffalo, NY, United States; ^4^Realm of Caring Foundation, Colorado Springs, CO, United States; ^5^Canopy Growth Corporation, Smiths Falls, ON, Canada

**Keywords:** anxiety, depression, cannabis, CBD-cannabidiol, THC-tetrahydrocannabinol

## Abstract

**Background:** Anxiety and depressive disorders are highly prevalent. Patients are increasingly using medicinal cannabis products to treat these disorders, but little is known about the effects of medicinal cannabis use on symptoms of anxiety and depression. The aim of the present observational study was to assess general health in medicinal cannabis users and non-using controls with anxiety and/or depression.

**Methods:** Participants (368 Cannabis Users; 170 Controls) completed an online survey assessing anxiety and depressive symptoms, cannabis product use, sleep, quality of life, and comorbid chronic pain. Participants that completed this baseline survey were then invited to complete additional follow-up surveys at 3-month intervals. Baseline differences between Cannabis Users and Controls were assessed using independent-samples *t*-tests and generalized linear mixed effects models were used to assess the impact of initiating cannabis product use, sustained use, or discontinuation of use on anxiety and depressive symptoms at follow-up.

**Results:** Medicinal cannabis use was associated with lower self-reported depression, but not anxiety, at baseline. Medicinal cannabis users also reported superior sleep, quality of life, and less pain on average. Initiation of medicinal cannabis during the follow-up period was associated with significantly decreased anxiety and depressive symptoms, an effect that was not observed in Controls that never initiated cannabis use.

**Conclusions:** Medicinal cannabis use may reduce anxiety and depressive symptoms in clinically anxious and depressed populations. Future placebo-controlled studies are necessary to replicate these findings and to determine the route of administration, dose, and product formulation characteristics to optimize clinical outcomes.

## Introduction

Anxiety and depressive disorders are highly prevalent (1), recurrent (2, 3), and can have a substantial negative impact on quality of life (4). Outcomes are worsened in the likely incidence of comorbidity (5–7), and both depression and comorbid anxiety/depression are associated with increased risk of mortality (8), particularly in people with co-occurring chronic physical illnesses (9). Yet, treatment is often not pursued despite the availability of multiple treatment options (6, 10–13).

Several pharmacotherapeutic interventions show efficacy in the treatment of anxiety and depression (14, 15). However, many patients are skeptical about the use of medication (16, 17), and antidepressants, the most frequently prescribed of these medications (18, 19), are not without contraindications. Adverse events are fairly common across antidepressant drug classes, and can disincentivize initiation and contribute to discontinuation of pharmacotherapy (14, 20–24). Further, although antidepressants are demonstrably superior to placebo at alleviating symptoms of both anxiety and depression, effect sizes are small (14, 25), and, thus, may not always be perceptible at the patient level. Finally, discontinuation of antidepressant treatment after sustained use is associated with a withdrawal syndrome in most patients that ranges in severity and can last for several months (26). Taken together, even though there is clear evidence of efficacy for antidepressants at the population level, perceived variability in cost-benefit ratio at the patient level means many people with anxiety or depression are interested in alternative options.

In this vein, an increasing number of people struggling with anxiety and/or depression are trying cannabis products for symptom management (27–29). Cannabis products can generally be separated into three “chemotypes” based on the predominant chemical constituents: (1) Δ9-tetrahydrocannabinol (THC) dominant products, (2) cannabidiol (CBD) dominant products, and (3) products that contain roughly equal amounts of both THC and CBD. Published studies on the impact of cannabis use on anxiety and depression have shown mixed results, and often vary based on the chemotype of the product under investigation and the duration of the dosing regimen. For example, the two studies in which THC was acutely administered to people with clinical anxiety showed limited evidence of anxiolysis (30, 31), but chronic nabilone (oral synthetic THC analog) administration over 4 weeks was associated with a significant reduction in anxiety in a placebo-controlled trial (32). Differential effects of cannabinoid type 1 receptor (CB1R) agonists, like THC, observed in long-term vs. acute dosing studies may be a product of increased CB1R binding on cortical glutamatergic neurons due to CB1R downregulation on GABAergic terminals (33, 34); a similar mechanism is implicated in the dose-dependent effects of acute THC exposure on anxiety (33). Neuroplasticity following extended exposure may also explain anxiogenesis frequently reported in cannabis withdrawal (35).

Effects of THC treatment on depression also appear mixed, though no clinical trials have been conducted to examine a direct effect of THC on depressive symptoms. Epidemiological studies suggest that non-medicinal (“recreational”) use of cannabis, which is typically THC-dominant, may be associated with increased risk of developing a depressive disorder (36) and greater depressive symptom severity (37), an association not observed for anxiety disorders (38). Rather than being causative, however, non-medicinal cannabis use may instead represent an attempt at self-medication during a prodromal period. Indeed, the CB1R agonist activity of THC mimics endogenous cannabinoid signaling, which is notably downregulated in women with clinical depression (39), and endogenous cannabinoids appear to regulate neural serotonergic signaling (40). THC itself can produce feelings of euphoria (41), and clinical trials of a CB1R antagonist were discontinued following reported increases in depression and suicidality (42). However, THC has shown no evidence of antidepressant efficacy when assessed as a secondary outcome in treatment trials for chronic pain, though self-reported depression scores in these trials were already low at baseline (43–45).

CBD, a phytocannabinoid that lacks the abuse liability of THC (46), has potential for therapeutic use in psychiatry. CBD has shown anxiolytic efficacy both acutely [(47, 48) but see (31)] and following chronic treatment in people with clinical anxiety (49). Preclinical evidence suggests that anxiolytic effects are produced *via* 5-HT1A receptor agonism in both acute (50–52) and chronic dosing models, without impacting 5-HT1A receptor expression (53). This lack of neural remodeling may explain why CBD discontinuation does not appear to produce a THC-like withdrawal syndrome (54). Additionally, the proposed serotonergic mechanism of CBD is distinct from that used by most common antidepressant medications, which selectively inhibit cellular reuptake of serotonin and/or norepinephrine (SSRIs, SNRIs), and is instead more comparable to the anxiolytic medication buspirone (55). Like CBD, buspirone does not appear to produce a withdrawal syndrome (56). Antidepressant effects of CBD have also been consistently demonstrated preclinically following both acute and chronic administration (57–60), though no clinical trials have yet been published. Antidepressant effects appear to be a product of the same serotonergic mechanism that drives anxiolysis (57, 58), and have been shown to synergize with other serotonergic medications (61). This again draws comparison with buspirone, which shows evidence of efficacy both as a depression monotherapy (62) or as an adjunct treatment to SSRIs (63).

Research evaluating the anxiolytic or antidepressant effects of products with a more balanced THC:CBD ratio is limited. Some studies in humans indicate that concurrent CBD/THC administration attenuates anxiogenic effects produced by THC (64, 65), but this has not been observed consistently (66, 67) and may be dose-dependent (68). This inconsistency is mirrored in the preclinical literature (69–71), making it difficult to determine a responsible mechanism given the diverse pharmacological activity of CBD (72, 73). Balanced THC:CBD products have also not been assessed for efficacy in psychiatric populations, though effects on anxiety and depression have been reported as secondary outcomes in clinical trials for other conditions. Nabiximols produced no effect on symptoms of anxiety or depression in people with multiple sclerosis (74) or in people with chronic pain due to cancer (75). Notably, both of these studies listed current psychiatric diagnosis as exclusion criteria, making it difficult to extrapolate these outcomes to people with clinical anxiety or depression.

Given these conflicting outcomes, the impact of medicinal cannabis use on anxiety and depression remains an open question. Our group previously found that medicinal cannabis users reported reduced anxiety and depression when compared to a control group that was considering, but had not yet initiated medicinal cannabis use (76). This impact of cannabis use was observed both cross-sectionally and longitudinally. However, effect sizes were only modest, and this was likely a product of the diverse array of clinical conditions represented in the sample. The purpose of the current study was to extend prior findings by limiting our focus to only participants that reported having anxiety and/or depression. We also expand on other epidemiological work that has principally focused on the impact of non-medicinal cannabis use on symptoms of anxiety and depression by providing insight into the effects of medicinal cannabis use on these symptoms.

## Materials and Methods

### Study Design

The analyses herein represent a subsample of participants that were enrolled in the parent study between April 2016 and July 2020 (76). Briefly, the parent study was a collaboration between Johns Hopkins University School of Medicine (Baltimore, MD) and the Realm of Caring Foundation (Colorado Springs, CO), and comprised a series of online surveys (Qualtrics, Provo, UT). Participants were recruited from the Realm of Caring patient registry and *via* social media advertisements. Those interested in participating were provided with a unique identification number, a study overview, and instructions for survey completion. Those interested in enrolling provided informed consent before beginning the baseline survey. Upon completion of the baseline survey, participants were invited to complete additional follow-up surveys at 3-month intervals. Compensation for completing each survey was entry into a monthly drawing for a $50 gift card. All procedures were approved by the Johns Hopkins IRB.

### Participants

Study participants were people that completed the baseline survey and reported having anxiety and/or depression (*n* = 538). Participants were included if they were at least 18 years old and endorsed having major depressive disorder, postpartum depression, dysthymia, premenstrual dysphoric disorder, seasonal affective disorder, generalized anxiety disorder, panic disorder, social anxiety disorder, and/or agoraphobia. Participants that did not list a specific disorder and instead only endorsed “anxiety” or “depression” were also included. Of this sample, *n* = 368 participants reported current use of medicinal cannabis products at baseline (“Cannabis Users”), and *n* = 170 were considering the use of medicinal cannabis, but had not yet initiated use (“Controls”). Follow-up assessments were offered every 3 months after enrollment until the study closed in July 2020, and participants could complete as many as desired. Of the participants that completed the baseline survey, *n* = 211 completed at least one follow-up assessment (*n* = 145 Cannabis Users; *n* = 66 Controls), and the average number of completed assessments for these participants was 2.2 (median = 1). Follow-up assessments were recorded at an average of 14 months (SD = 9) post-baseline, and the longest follow-up occurred 44 months after baseline. All follow-up assessments completed were included in longitudinal analyses.

### Outcome Measures

Surveys included validated self-report questionnaires and investigator-developed measures of health outcomes. Participants self-reported demographic information and any current medical condition(s). Medicinal cannabis use was evaluated using both multiple choice and free-response items pertaining to current use of medicinal cannabis, cannabis product type (e.g., dried flower, hemp extract oil), chemotype (e.g., THC-dominant, CBD-dominant, and balanced THC:CBD ratio), product dosing regimen, and product route of administration. Information pertaining to current use of prescription medication(s) was also collected.

Current symptoms of depression and anxiety were evaluated using the Hospital Anxiety and Depression Scale (HADS) (77), in which a score ≥ 8 on either subscale indicates clinical concern. The abbreviated version of the World Health Organization Quality of Life assessment (WHOQOL-BREF) was used to assess perceived quality of life, health satisfaction, and mood (78). Sleep was assessed using the Pittsburgh Sleep Quality Index (PSQI) (79), as sleep dysfunction is a symptom of both anxiety and depressive disorders (80). Consistent with epidemiological reports (81), chronic pain disorders were highly prevalent in this sample. Thus, recent pain was also assessed using the Numeric Pain Rating Scale (NPRS) (82). The free-response question “How has the therapeutic use of cannabis/cannabinoids harmed the participant?” provided participants with the opportunity to disclose any adverse effects of cannabis use.

### Analyses

Descriptive statistics were used to summarize participant demographics, cannabis use patterns, and qualitative effects of cannabis use. Demographic differences by cannabis use were evaluated using independent samples *t*-tests (continuous measures) or Fisher's exact test (dichotomous measures). Independent-samples *t*-tests were used to assess baseline differences between Cannabis Users and Controls on the depression and anxiety subscales of the HADS, overall sleep quality score on the PSQI, past-month average and worst pain on the NPRS, and components of the WHOQOL-BREF. Independent-samples *t*-tests were used for these baseline, cross-sectional analyses given the comparison of two independent groups (Cannabis User vs. Control). Logistic regression was used to compare baseline group differences in possible clinical cases based on the HADS anxiety and depression cutoff scores (≥8). Additional 2-way ANOVAs were conducted for anxiety and depression scores evaluating interactions between cannabis product use and participant gender, cannabis product use and concomitant use of serotonergic medications, and cannabis product use and psychiatric subtype (Anxiety Only, Depression Only, or Both). As many participants reported use of multiple cannabis product chemotypes, chemotype differences were evaluated using independent-samples *t*-tests comparing (1) Cannabis Users who did vs. did not use CBD-dominant products and (2) did vs. did not use THC-dominant products. Baseline associations between HADS depression and anxiety scores with past-month average pain on the NPRS were assessed using Pearson correlations.

For longitudinal data, generalized linear mixed effect models were used to evaluate changes over time in HADS subscale scores and the Psychological domain of the WHOQOL-BREF for three groups (1) baseline Controls who initiated cannabis use (“Initiators”; *n* = 36), (2) baseline Controls who did not initiate cannabis use (“Non-initiators”; *n* = 23), and (3) baseline Cannabis Users who continued use (“Sustainers”; *n* = 121). Participants that either discontinued cannabis use (*n* = 10) or alternated between use and non-use across follow-up assessments (*n* = 21) were not analyzed due to small sample size and to maintain consistency in analyses, respectively. Generalized linear mixed effect models were used to account for the repeated measurement over time, inclusion of participants with missing data, and inclusion of continuous predictors. Statistical tests evaluated if changes over time differed by group (Group × Time interactions) with within-group tests conducted for significant interactions. Missing data were treated as missing at random and addressed using full-information-maximum-likelihood estimation procedures given evidence that attrition in follow-up was not significantly related to baseline anxiety or depression scores (see Discussion). All tests were conducted as two-tailed tests with an initial alpha level of 0.05. A Bonferroni correction was then used to adjust for multiple comparisons in cross-sectional analyses, setting the new alpha level for these at 0.00132 (0.05/38). Analyses were conducted in *R*.

## Results

### Demographics

Participants were mostly female (79%), Caucasian (83%), and had a mean age of 46 years old (SD = 13) at baseline (Table 1). Participants predominantly reported having comorbid anxiety and depression (51%), followed by anxiety alone (34%), and depression alone (15%). Most participants reported having a co-occurring chronic pain disorder (69%). Just over one-third of participants reported use of serotonergic medication(s) to treat depression and/or anxiety (36%) (Table 2 details serotonergic medication and doses). Fewer Cannabis Users endorsed serotonergic medication use compared with Controls [OR = 0.49, *p* < 0.001]. No other differences were observed between groups (all *p* > 0.17).

**Table 1:** Demographics.

	**Cannabis Users (*n* = 368)**	**Controls (*n* = 170)**	***p***
Age, Mean (SD)	46 (13)	46 (12)	0.96
Women, *n* (%)	286 (78%)	141 (83%)	0.17
White, *n* (%)	298 (85%)	148 (89%)	0.34
Post-secondary degree, *n* (%)	216 (60%)	107 (63%)	0.50
Psychiatric condition			0.28
Anxiety only, *n* (%)	134 (36%)	50 (29%)	
Depression only, *n* (%)	54 (15%)	27 (16%)	
Both anxiety and depression, *n* (%)	180 (49%)	93 (55%)	
Use of serotonergic medication, *n* (%)	116 (33%)	80 (51%)	<0.001
Comorbid chronic pain, *n* (%)	250 (68%)	121 (71%)	0.48

*Percentages calculated for participants without missing data on that item*.

**Table 2:** Use of serotonergic medication.

	**Median daily dose in mg (range; % taking)**	
	**Cannabis users (*n* = 116)**	**Controls (*n* = 80)**
Duloxetine	60 (20–120; 21%)	60 (30–120; 19%)
Sertraline	100 (25–200; 19%)	100 (12.5–150; 20%)
Venlafaxine	150 (37.5–300; 10%)	150 (37.5–300; 21%)
Fluoxetine	40 (10–150; 13%)	40 (10–80; 18%)
Escitalopram	20 (1.5–20; 16%)	20 (5–30; 14%)
Trazodone	100 (37.5–125; 9%)	50 (50–200; 10%)
Buspirone	35 (30–60; 7%)	17.5 (5–30; 4%)
Mirtazapine	15 (7.5–30; 7%)	22.5 (15–30; 4%)
Citalopram	40 (10–60; 5%)	20 (20–40; 4%)
Paroxetine	40 (20–40; 3%)	30 (20–40; 3%)
Desvenlafaxine	75 (50–150; 3%)	50 (50; 1%)
Vortioxetine	10 (10; 2%)	10 (10; 1%)
Vilazodone	20 (20; 1%)	–
Levomilnacipran	–	– (–, 1%)

*Twenty-nine participants (n = 16 Cannabis Users; n = 13 Controls) reported use of multiple medications*.

### Cannabis Product Use

Ninety-five participants (26%) endorsed medicinal cannabis use, but did not know the cannabinoid content of the product(s). Among Cannabis Users that did know the chemotype of product(s) they used, most reported use of CBD-dominant products (82%), followed by THC-dominant (23%), balanced THC:CBD (7%), and products for which the highest concentration was a “minor cannabinoid” [e.g., cannabigerol (CBG), cannabinol (CBN); 5%]. Nearly one-third of participants (*n* = 122) reported use of multiple cannabis product types, including products with unknown chemical composition. These participants were counted as contributing to each of the reported chemotype categories in the distributions and in analyses of medicinal cannabis efficacy by chemotype. For example, participants that indicated concurrent use of both CBD-dominant and THC-dominant products were counted for both the 82 and 23% figures reported above. Most participants that used THC-dominant products also used a CBD-dominant product (83%), but only 24% of participants that used a CBD-dominant product also used a THC-dominant product.

A subset of study participants (*n* = 139) reported use of specific CBD-dominant products for which certificates of analysis were obtained from manufacturers in order to calculate total daily CBD/THC dose. The mean oral CBD daily dose reported was 61 mg (median = 30 mg; range = 0.4–1,050 mg) and the mean oral THC dose was 2.1 mg (median = 1 mg; range ≤ 0.01–40.3 mg). Mean daily doses were 0.8 mg/kg CBD (median = 0.46 mg/kg; range ≤ 0.01–10.1 mg/kg) and 0.03 mg/kg THC (median = 0.02 mg/kg; range ≤ 0.01–0.39 mg/kg) when adjusted for body weight.

### Depression and Anxiety

**Figure 1:**
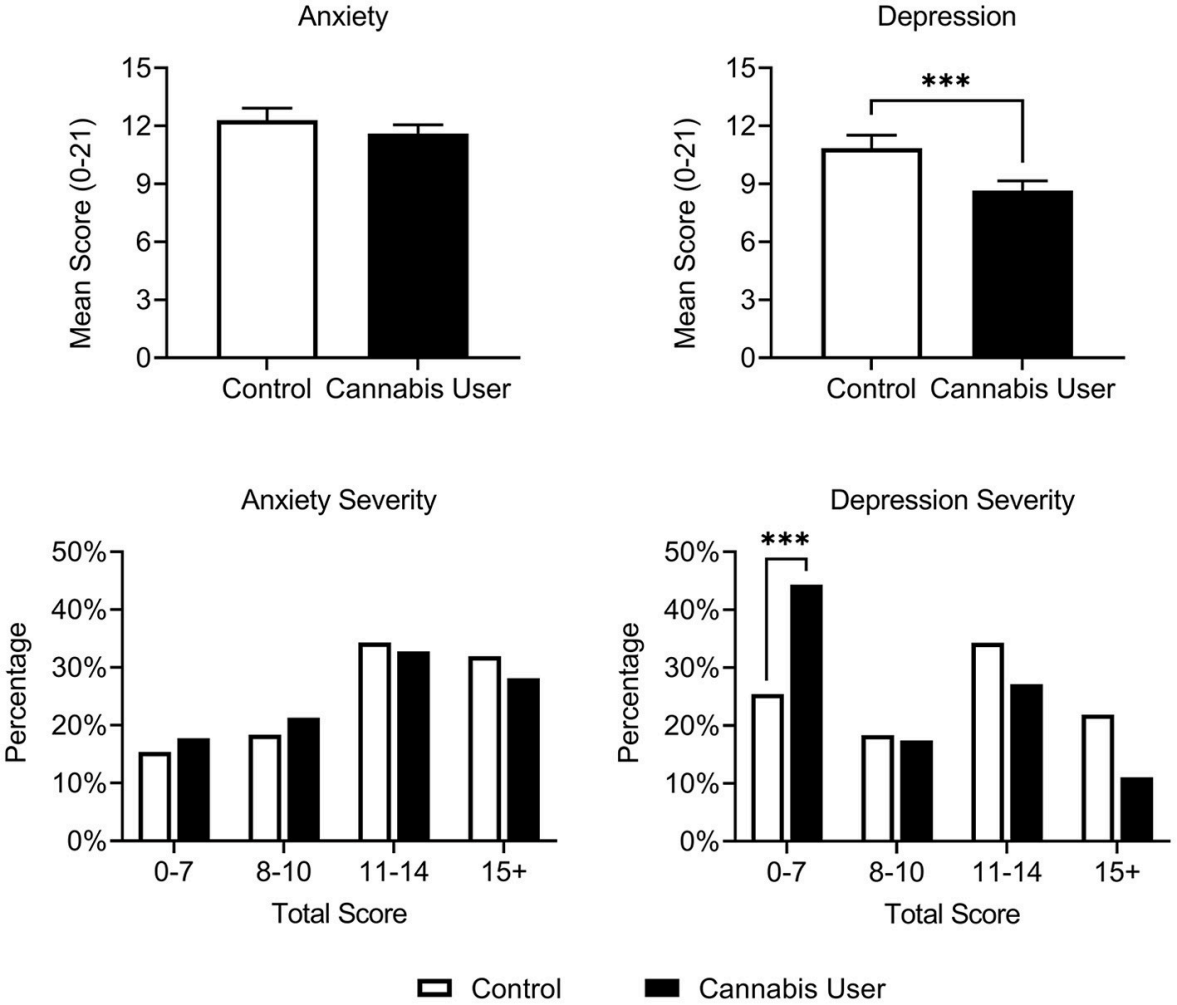
Cannabis Users (*n* = 368) had reduced depression, but not anxiety, relative to Controls (*n* = 170) on the HADS at baseline. A greater proportion of Cannabis Users also scored below the HADS cutoff for clinical concern (scores ≥ 8) relative to Controls. Scores ranging from 8 to 10, 11 to 14, and 15 to 21 represent approximate cutoffs for mild, moderate, and severe cases, respectively (83). ***p < 0.001.

Cannabis Users reported lower baseline depression [*t*_(528)_ = 4.995, *p* < 0.001, and *d* = 0.47], but not anxiety [*t*_(533)_ = 1.686, *p* = 0.09, *d* = 0.16], on the HADS compared with Controls (Figure 1). Cannabis Users were also more likely to present below the HADS cutoff for clinical concern (scores ≥ 8) for depression [OR = 2.33, *p* < 0.001], but not anxiety [OR = 1.19, *p* = 0.50]. These findings remained after correcting for multiple comparisons. No interactions were observed between cannabis use and participant gender, psychiatric subtype, or use of serotonergic medication on anxiety or depression scores (all *p* > 0.07).

### Sleep

Cannabis Users reported significantly better past-month sleep quality than Controls at baseline on the PSQI [*t*_(463)_ = 3.209, *p* = 0.001, and *d* = 0.32]. This remained significant after correction for multiple comparisons.

### Quality of Life

Cannabis Users rated their overall quality of life more highly than Controls at baseline [*t*_(525)_ = −3.327, *p* < 0.001, and *d* = 0.31], reported greater health satisfaction [*t*_(525)_ = −4.248, *p* < 0.001, and *d* = 0.40], and had higher Psychological domain scores [*t*_(524)_ = 5.35, *p* < 0.001, and *d* = 0.50] on the WHOQOL-BREF. These findings remained significant after correction for multiple comparisons.

### Pain

Cannabis Users reported lower past-month average pain at baseline relative to Controls [*t*_(527)_ = 3.281, *p* = 0.001, and *d* = 0.31], but no difference was observed in worst pain on the NPRS [*t*_(525)_ = 0.987, *p* = 0.324, and *d* = 0.09]. Average pain was positively correlated with HADS depression scores in both Cannabis Users [*r* = 0.4, *p* < 0.001] and Controls [*r* = 0.37, *p* < 0.001], consistent with prior research in people with chronic pain (84). Average past-month pain was also positively correlated with HADS anxiety scores in Cannabis Users, albeit to a lesser extent [*r* = 0.25, *p* < 0.001], and uncorrelated in Controls [*r* = 0.1, *p* = 0.18]. These findings remained the same after correction for multiple comparisons.

### Chemotype

Comparing Cannabis Users based on chemotype indicated that CBD-dominant product use was associated with lower HADS depression scores [*t*_(359)_ = 2.609, *p* = 0.009, and *d* = 0.36], improved quality of life [*t*_(358)_ = 2.849, *p* = 0.005, and *d* = 0.39], and higher Psychological domain scores on the WHOQOL-BREF [*t*_(357)_ = 2.02, *p* = 0.04, and *d* = 0.27] compared to non-use of CBD products. These findings were no longer significant after correction for multiple comparisons. Outcomes did not differ in Cannabis Users based on use vs. non-use of THC dominant products (all *p* > 0.05; all *d* < 0.25).

### Adverse Events

In response to the question “How has therapeutic use of cannabis harmed the participant?,” the majority of Cannabis Users reported no perceived harms (61%) or left this question blank (14%). Harms that were reported included high cost (7%), social stigma or legal issues (5%), intoxication (2%), unpleasant effects associated with inhalation (e.g., smell of smoke, worsening asthma; 2%), impaired cognition (2%), fatigue (2%), and gastrointestinal problems or nausea (1%). Ten participants (3%) reported that medicinal cannabis worsened symptoms of anxiety or caused paranoia, and one participant (< 1%) reported that it worsened symptoms of depression. Five percent of cannabis users reported other unique harms (i.e., for which they represented an *n* of 1).

The incidence of adverse events differed by chemotype. Though no differences were observed between CBD and non-CBD users, a significantly greater proportion of THC users that responded to this question reported an adverse event relative to non-THC users [38 vs. 26%; *p* = 0.04]. A higher percentage of THC users reported intoxication [6 vs. 2%; *p* = 0.04], unpleasant effects associated with inhalation [6 vs. < 1%; *p* = 0.004], and worsened symptoms of anxiety or paranoia [8 vs. 2%; *p* = 0.02] relative to non-THC users. A greater proportion of THC users also reported harms involving social stigma or legal issues compared to non-THC users [12 vs. 3%; *p* = 0.009]. These findings were no longer significant after correction for multiple comparisons.

### Longitudinal Impact on Depression and Anxiety

**Figure 2:**
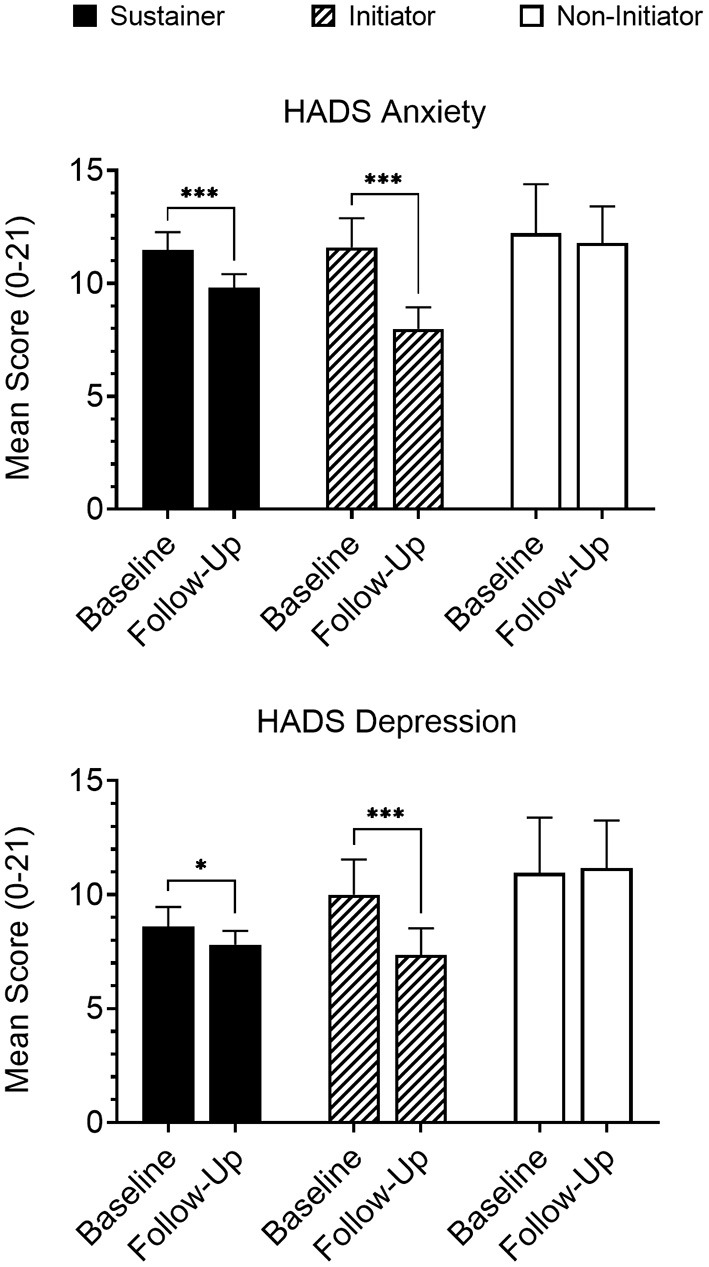
Participants that either initiated medicinal cannabis use (“Initiators”; *n* = 36) or continued cannabis use that had been reported at baseline (“Sustainers”; *n* = 121) reported significantly reduced anxiety and depression on the HADS at follow-up, while participants that did not initiate cannabis use (“Non-initiators”; *n* = 23) reported no change. Follow-up data presented are collapsed across all completed follow-up assessments for members of each group. *p < 0.05; ***p < 0.001.

An interaction was observed between timepoint (baseline vs. follow-up) and longitudinal group (Initiator, Sustainer, Non-initiator) for both HADS anxiety [*p* = 0.04] and depression [*p* = 0.009] scores, and for Psychological domain scores on the WHOQOL-BREF [*p* = 0.02]. Within-group analyses indicated that Initiators reported a significant reduction in both mean anxiety [*b* = −2.52, *p* < 0.001] and depression [*b* = −2.57, *p* < 0.001] scores from baseline to follow-up assessments (i.e., improved symptoms; Figure 2); improvement was also observed for Psychological domain scores [*b* = 1.39, *p* < 0.001]. This effect was observed to a lesser extent in Sustainers for HADS anxiety [*b* = −1.40, *p* < 0.001] and depression [*b* = −0.65, *p* = 0.03] and insignificantly in the Psychological domain [*b* = 0.33, *p* = 0.07]. Non-initiators did not report changes in HADS anxiety [*b* = −0.25, *p* = 0.67], depression [*b* = −0.67, *p* = 0.24], or Psychological domain [*b* = 0.42, *p* = 0.25] scores during the study.

Evaluation of clinical cutoffs revealed similar results wherein Initiators showed greater odds of going below clinical cutoffs (scores ≥ 8) (77) at follow-up for HADS anxiety [OR = 14.07, *p* = 0.002] and depression [OR = 6.47, *p* = 0.01] scales, Sustainers did to a smaller extent for both anxiety [OR = 3.79, *p* = 0.001] and depression [OR = 2.56, *p* = 0.02], but Non-initiators did not show a significant change in odds for either anxiety [OR = 0.03, *p* = 0.33] or depression [OR = 1.63, *p* = 0.66].

## Discussion

Despite the high incidence of anxiety and depressive disorders, particularly among women (1), many people suffering with these conditions do not pursue treatment (6, 10–13) and pharmacotherapeutic options remain subpar (14, 23, 25). Here, we evaluated symptoms of anxiety and depression, as well as other general health metrics, in a convenience sample of medicinal Cannabis Users with anxiety and/or depression in comparison with a non-using Control group in a longitudinal web-based survey study. In the cross-sectional comparison at baseline, Cannabis Users self-reported less severe depression, but not anxiety. This effect was strongest among participants using CBD-dominant products, and was not impacted by participant gender or concurrent use of serotonergic antidepressants. Cannabis Users also reported superior sleep, quality of life, and lower average pain relative to Controls at baseline. Adverse effects attributed by participants to cannabis product use were infrequent, were more associated with THC-dominant product use, and, with the exception of nausea, were distinct from those typically associated with antidepressants (21, 23). In longitudinal analyses, participants who initiated medicinal cannabis use during the follow-up period showed a significant reduction in both depression and anxiety symptoms. A similar, albeit smaller magnitude, effect was observed in participants that sustained medicinal cannabis use throughout the study, suggesting an improvement in anxiety and depression symptoms with both the onset of cannabis use and with extended use.

A handful of studies have previously examined the anxiolytic effects of THC (30–32) and CBD (31, 47–49) in clinical populations, and most of these have found a positive effect. While we observed no effect of medicinal cannabis use on anxiety at baseline, participants that initiated cannabis use during the follow-up period reported a significant reduction in anxiety that was not mirrored in Non-initiators. This discrepancy between cross-sectional and longitudinal anxiety outcomes may reflect on the therapeutic window for CBD. As participants may have been using medicinal cannabis for any period of time prior to baseline assessment to be considered a Cannabis User, differences in outcomes may be attributable to the development of tolerance to anxiolytic effects of cannabinoids, although positive effects observed in the Sustainer group suggest otherwise. Alternatively, this discrepancy may reflect a latency period during which the clinical benefits of cannabinoids for anxiety are not yet observed. Controlled clinical studies and dense sampling data (e.g., ecological momentary assessment) are needed to clarify these early-in-treatment effects with greater precision. It is also important to note that the CBD doses used in trials that found an anxiolytic effect were far greater than the average reported by participants in our study, and the time course for anxiolytic efficacy may differ by dose (47–49). Finally, it is possible that anxiolytic effects of CBD may be condition-specific, as symptom improvement has been consistently observed in studies of social anxiety disorder (47–49), but not in obsessive compulsive disorder (31). The heterogeneity of our sample and reliance on self-report for psychiatric condition(s) of a given participant may have precluded observation of anxiolysis at baseline.

Assessment of the antidepressant effects of medicinal cannabis use has thus far been more limited. In our study, we found that Cannabis Users reported reduced depression relative to non-using Controls at baseline. Consistent with prior research in people with comorbid chronic pain (43–45), we found that use of THC-dominant products was not superior to use of non-THC-dominant products in alleviating depression symptoms. In contrast, participants that reported use of CBD-dominant products provided significantly lower depression scores relative to those that did not, consistent with preclinical findings (57, 85, 86). Cannabis Users also reported superior sleep, average pain, and quality of life relative to Controls. This is unsurprising given the interrelated nature of these constructs with depression (87–89), and both pain and quality of life have been shown to be improved with traditional antidepressant treatment (90, 91). Consistent with baseline outcomes, we found that initiation of medicinal cannabis use was associated with a significant reduction in depressive symptoms, sustained use was associated with a modest reduction, and participants that did not use cannabis at all showed no difference in symptom expression between baseline and follow-up. These combined cross-sectional and longitudinal findings show a consistent antidepressant effect of medicinal cannabis.

This study has several limitations. Notably, these outcomes rely entirely on participant self-report. It is possible that some of the symptom improvement reported by medicinal Cannabis Users can be attributable to an expectancy effect, especially in consideration of the low daily CBD doses reported by participants relative to those used in previous clinical studies (31, 47–49). However, as research in this area is still in its infancy, it is presently unknown what constitutes an optimal dose to maximize antidepressant/anxiolytic efficacy and minimize incidence of side effects, particularly when dosing over an extended period. It is also unclear if minor cannabinoids or terpenes present in whole-plant or “full spectrum” products used in this study, compared with isolated pure chemical substances used in previous research, confer additional therapeutic benefits *via* an entourage effect (92). Similarly, Controls were people that were considering the use of medicinal cannabis to treat their condition. Therefore, it is also not surprising that people who believed medicinal cannabis might assist with their condition enough to initiate use would perceive a substantial benefit. This study additionally represents a convenience sample of people registered with the Realm of Caring foundation willing to complete lengthy online assessments for only modest incentives. People with mild symptoms, with more responsibilities in daily life, or who did not see a substantial improvement from medicinal cannabis treatment may have been less likely to complete the survey. Finally, dosing data could only be obtained for about half of the medicinal Cannabis Users given poor or non-existent labeling information on some retail and all black market cannabis products, and most participants did not complete a follow-up assessment. Supplemental analyses showed no significant differences in baseline health behavior between participants who did and did not provide follow-up data, *p* > 0.39, *d* < 0.08.

In sum, this study suggests that use of CBD-dominant cannabis products is associated with reduced depression in a sample of mostly female, Caucasian adults. The study extends prior research by including a control group, and through a study design that includes both cross-sectional and within-subject, longitudinal comparisons. Though antidepressant effects of CBD have been consistently reported preclinically (57, 85, 86), our work contributes to the literature by showing a potential for translation across species without many of the negative side effects associated with traditional antidepressants (21, 23). It is recommended that this antidepressant effect of CBD be evaluated further in placebo-controlled clinical trials, and that participants remain under observation following treatment completion to confirm the absence of a discontinuation syndrome. Additionally, considering the average daily CBD dose reported in our study was quite low relative to previous clinical work (31, 47–49), future research is necessary to determine best dosing practices to achieve optimal antidepressant effects. Medicinal cannabis products may also alleviate anxiety, but it is unclear if this effect is gated by duration of use. Placebo-controlled clinical trials are necessary to further explore the potential efficacy of CBD in the treatment of anxiety and depression.

## Data Availability Statement

The datasets presented in this article are not readily available because the corresponding author does not own this data. Requests to access the datasets should be directed to Ryan Vandrey (rvandrey@jhmi.edu).

## Ethics Statement

The studies involving human participants were reviewed and approved by Institutional Review Board of Johns Hopkins University. The patients/participants provided their written informed consent to participate in this study.

## Author Contributions

HJ, RV, MB-M, NS, and JM conceptualized and executed the parent survey. EM conceptualized this secondary analysis, curated the data, and drafted the original outline. EM and JS conducted statistical analyses and created the figures and tables. EM, JS, and RV wrote the first and final manuscript drafts. All authors reviewed and edited both manuscript drafts and approved the final manuscript.

## Funding

This work was supported by the Realm of Caring Foundation and by National Institute on Drug Abuse (NIDA) grants T32-DA007288 (EM) and T32-DA007209 (NS and JS).

## Conflict of Interest

RV has received financial compensation as a consultant or advisory board member from Canopy Growth Corporation, MyMD Pharmaceuticals, and Syqe Medical Ltd. MB-M is an employee of Canopy Growth Corporation and past board of directors member for AusCann Group Holdings Ltd. The remaining authors declare that the research was conducted in the absence of any commercial or financial relationships that could be construed as a potential conflict of interest.

## Publisher's Note

All claims expressed in this article are solely those of the authors and do not necessarily represent those of their affiliated organizations, or those of the publisher, the editors and the reviewers. Any product that may be evaluated in this article, or claim that may be made by its manufacturer, is not guaranteed or endorsed by the publisher.
